# YAP as a key regulator of adipo-osteogenic differentiation in human MSCs

**DOI:** 10.1186/s13287-019-1494-4

**Published:** 2019-12-18

**Authors:** Chanchao Lorthongpanich, Kanjana Thumanu, Kantpitchar Tangkiettrakul, Nittaya Jiamvoraphong, Chuti Laowtammathron, Nattaya Damkham, Yaowalak U-pratya, Surapol Issaragrisil

**Affiliations:** 1 Siriraj Center of Excellence for Stem Cell Research, Department of Medicine, Faculty of Medicine Siriraj Hospital, Mahidol University, 2 Wanglang Road, Bangkoknoi, Bangkok, 10700 Thailand; 2 Synchrotron Light Research Institute (Public Organization), Nakhon Ratchasima, Thailand; 3 Department of Immunology, Faculty of Medicine Siriraj Hospital, Mahidol University, Bangkok, Thailand; 4 Division of Hematology, Department of Medicine, Faculty of Medicine Siriraj Hospital, Mahidol University, Bangkok, Thailand; 5 Bangkok Hematology Center, Wattanosoth Hospital, BDMS Center of Excellence for Cancer, Bangkok, Thailand

**Keywords:** FTIR microspectroscopy, Dobutamine, LPA, MSCs, Adipo-osteogenic balance, YAP, Hippo pathway

## Abstract

**Background:**

Mesenchymal stem cells (MSCs) are multipotent stem cells that are able to differentiate into several cell types, including cartilage, fat, and bone. As a common progenitor, MSC differentiation has to be tightly regulated to maintain the balance of their differentiation commitment. It has been reported that the decision process of MSCs into fat and bone cells is competing and reciprocal. Several factors have been suggested as critical factors that affect adipo-osteogenic decision, including melatonin and smad4. Yes-associated protein (YAP) is an important effector protein in the Hippo signaling pathway that acts as a transcriptional regulator by activating the transcription of the genes involved in cell proliferation and anti-apoptosis. The non-canonical role of YAP in regulating bone homeostasis by promoting osteogenesis and suppressing adipogenesis was recently demonstrated in a mouse model. However, it is unclear whether YAP is also crucial for modulating human MSC differentiation to fat and bone.

**Methods:**

The expression level of YAP during MSC differentiation was modulated using pharmaceutical molecule and genetic experiments through gain- and loss-of-function approaches.

**Results:**

We demonstrated for the first time that YAP has a non-canonical role in regulating the balance of adipo-osteogenic differentiation of human MSCs. The result from synchrotron radiation-based Fourier transform infrared (FTIR) microspectroscopy showed unique metabolic fingerprints generated from YAP-targeted differentiated cells that were clearly distinguished from non-manipulated control.

**Conclusions:**

These results, thus, identify YAP as an important effector protein that regulates human MSC differentiation to fat and bone and suggests the use of FTIR microspectroscopy as a promising technique in stem cell research.

## Background

Mesenchymal stem cells/stromal cells (MSCs) are a specialized population of progenitor cells that can be isolated from various adult or fetal tissues and membranes [1]. Due to their hypoimmunogenic or immune privilege, MSCs have been used in regenerative therapy, especially for transplantation across major histocompatibility barriers [2, 3]. MSCs are multipotent stem cells that are able to differentiate into several cell types, including osteoblasts, chondrocytes, adipocytes, and hematopoietic stem cell-supportive stroma. As a common progenitor, MSCs have to maintain a delicate balance for their differentiation commitment, especially for differentiation to fat and bone [4]. It has been demonstrated that bone induction factors, such as RUNX family transcription factor 2 (Runx2), inhibit adipogenesis, whereas peroxisome proliferator-activated receptor γ (PPARγ) stimulated adipogenesis and inhibited osteogenesis [5]. Bone loss has also been observed in obese mice, rats, and humans [6–8]. Several external cues contribute to the bias of adipo-osteogenic differentiation of MSCs, including chemical [9, 10], physical [11, 12], and biological factors, such as aging/metabolism [13]. These factors trigger different signaling pathways and activate various transcription factors that guide MSCs to commit to their differentiation fate [14]. The factors affecting the adipo-osteogenic decision of MSCs are reviewed in detail elsewhere [4].

It was recently observed that activation of the beta-1 adrenergic signaling pathway by dobutamine hydrochloride (DH), a beta-1 adrenergic agonist, contributes to postmenopausal and age-related bone loss, while blocking of beta-1 adrenergic signaling showed favorable effects on bone turnover [15]. However, the insight molecular mechanism for these phenomena has not been described. Bao and colleagues showed that DH is able to attenuate yes-associated protein (YAP), which is a transcriptional coactivator that is negatively regulated by the Hippo signaling pathway, by inhibiting its nuclear translocation [16]. YAP and transcriptional co-activator with PDZ-binding motif (TAZ), also known as WW domain-containing transcriptional regulator 1 (WWTR1), were recently suggested as key regulators of bone homeostasis in mice by promoting osteogenesis and suppressing adipogenesis via the Smad4 or beta-catenin signaling pathway [17–19]. However, whether or not YAP plays a role in controlling the adipo-osteogenic balance of human MSCs has never been reported.

Lysophosphatidic acid (LPA) is a phospholipid derivative that can act as a signaling molecule to activate G protein-coupled receptors that are known to regulate cell proliferation and migration. Recent experiments suggested that the Hippo-YAP/TAZ signaling pathway is a downstream target of LPA for regulating cell proliferation and migration. It has been shown that LPA inhibits large tumor suppressor (LATS) kinase, a major core kinase of the Hippo-YAP/TAZ pathway, resulting in activation of YAP transcription coactivator and the expression of its downstream target genes. Though studies in monocytes and CV-1 cells (kidney fibroblast cell line derived from an African green monkey) suggested that LPA could bind to and activate PPARγ (which is a transcription factor also known to play a crucial role in adipogenesis), experiments in mouse preadipose cell line (3T3F442A) showed negative effect of LPA on adipogenic differentiation, as demonstrated by a reduction in the PPARγ-sensitive genes phosphoenolpyruvate carboxykinase (PEPCK) and adipocyte lipid-binding protein (ALBP) [20]. However, the role of LPA in human MSC differentiation to adipocytes has never been demonstrated.

Synchrotron-based Fourier-transform infrared spectroscopy (FTIR) microspectroscopy is a valuable tool for characterizing and describing the biochemical changes in cells and tissues. The positions of the peaks observed in the FTIR spectra provide significant information relative to the biochemical content of the macromolecules in the cells. Therefore, this information can be used as a fingerprint of the structure and functionality of specimens. The content of these molecules can also be linked to the stage of differentiation and the physiological state of cells [21–25]. FTIR microspectroscopy is a proven label-free method for studying a small sample size with high spatial resolution [26]. With the brightness of SR light, FTIR microspectroscopy can be used to study single cells with a good signal-to-noise ratio [27, 28].

In the present work, we studied the role of YAP in regulating the adipo-osteogenic balance in human MSCs. The expression level of YAP during MSC differentiation was manipulated using the pharmaceutical molecule (DH and LPA to inhibit and activate YAP, respectively) and a genetic approach (Crispr/Cas9 targeting YAP and YAPs5a to inhibit and overexpress YAP, respectively). In addition to other standard procedures that are used to confirm the presence of osteoblasts and adipocytes, FTIR microspectroscopy was used to track the structural changes of nucleic acids, proteins, and lipids in differentiated cells after YAP had been manipulated. The results showed that YAP plays an important role in controlling adipo-osteogenesis in human MSCs. It is clear that YAP is required for MSCs to achieve osteogenic fate, whereas the absence of YAP promotes adipogenic differentiation. This result also strongly suggests the use of FTIR is an effective method for studying cell characteristics of human osteogenesis and adipogenesis.

## Materials and methods

### Isolation and culture of hUC-MSCs

Three umbilical cords (UC) were obtained, cut into small pieces, and incubated with 0.25% (w/v) trypsin-EDTA (GIBCO™; Invitrogen Corporation, Carlsbad, CA, USA) for 30 min at 37 °C. Cell suspensions were collected and washed with phosphate-buffered saline (PBS) before being resuspended with culture medium, which consisted of Dulbecco’s modified Eagle’s medium (DMEM)-low glucose (Gibco®) supplemented with 10% fetal bovine serum (FBS; Merck Millipore, Burlington, MA, USA), and plated in culture vessels (Corning, Corning, NY, USA). Cultures were maintained at 37 °C in a humidified atmosphere containing 5% CO_2_. The culture medium was replaced every other day. Adherence cells were treated with 0.05% Trypsin-EDTA (Gibco®) and split to a seeding ratio of 5000 cells/cm^2^ for expansion.

### Immunophenotypical characterization

Primary culture from UC-MSCs (4 × 10^5^ cells) were resuspended in 50 μl of PBS and incubated with 10 μl of peridinin-chlorophyll proteins (PerCP), fluorescein isothiocyanate (FITC) or phycoerythrin (PE)-conjugated antibodies against CD45 (BioLegend, San Diego, CA, USA), CD34 (BioLegend), CD73 (BioLegend), CD90 (BioLegend), or CD105 (BioLegend) for 30 min at 4 °C in the dark. After washing with PBS, the cells were fixed with 1% paraformaldehyde (PFA). At least 10,000 labeled cells were acquired and analyzed using flow cytometry (FACSCanto™ or FACSCalibur™ analyzer; BD Biosciences, San Jose, CA, USA).

### Preparation of small molecules

Lysophosphatidic acid (LPA) and dobutamine hydrochloride (DH) were purchased from Sigma-Aldrich (St. Louis, MO, USA) and prepared as a 20 mmol/l stock solution in the diluent suggested by the manufacturer’s protocol. The final concentration of 10 μM of LPA and 20 μM of DH was used as a supplement into osteogenic and adipogenic differentiation media.

### Generation of YAP-targeted cells

Crispr/Cas9 plasmid construct targeting YAP was purchased from GenScript Corporation (Piscataway, NJ, USA). For the preparation of lentivirus, the plasmid was transfected into 4 × 10^6^ HEK293T cells in a 100-mm cell culture dish using Lipofectamine 3000 (Life Technologies, Carlsbad, CA, USA). After 24 h, the medium was changed to fresh HEK293T cell medium consisting of DMEM (Gibco®) + 10% FBS. Culture media were collected at day 2 after transfection, passed through a 0.45-μM filter (Jet Biofil, Guangzhou, China), and concentrated by transferring the virus-containing supernatant through Amicon Ultra-15 Centrifugal Filter Units (Merck Millipore), followed by centrifugation at 4000*g* for 30 min at 4 °C. The concentrated virus was collected and added to 5 × 10^4^ MSCs in the presence of 5 μg/ml polybrene (Sigma-Aldrich). The medium was changed the next day to completed media. The transfected cells were treated with 2 μg puromycin for 2 days to eliminate the non-transfected cells before being subjected to osteogenic and adipogenic differentiation.

### Generation of YAP-overexpressing cells

MSCs were transfected with plasmids to promote the overexpression of YAP using 4D nucleofector (Lonza, Basel, Switzerland). At 24 h after transfection, puromycin (2 μg) was added into the culture media for 2 days before the cells were subjected to osteogenic and adipogenic differentiation. Overexpression was confirmed by quantitative real-time polymerase chain reaction (RT-PCR).

### Quantitative PCR and data analysis

Isolated total RNA was reverse-transcribed using a High-Capacity cDNA Reverse Transcription Kit (Applied Biosystems, Foster City, CA, USA). Quantitative RT-PCR (qRT-PCR) was performed using Real-Time PCR Master Mix (Applied Biosystems) and the Universal Probe Library (UPL; Roche Life Science, Penzberg, Germany) in a final volume of 10 μl. RT-PCR assays were performed using a CFX384 Real-Time PCR System (Bio-Rad Laboratories, Hercules, CA, USA).

### Western blot analysis

The presence of YAP was determined by Western blotting. Total protein was extracted from cells using a cell lysis buffer (10× RIPA; Cell Signaling Technology, Danvers, MA, USA) containing protease inhibitors (Roche Life Science). The denatured protein was run onto 7% SDS/polyacrylamide gels, and the separated proteins were transferred to PVDF membranes (Merck Millipore) and probed with the following primary antibodies: anti-YAP, anti-phosphorylated YAP (Cell Signaling Technology) diluted 1:1000, and anti-β-actin peroxidase (ACTB; Sigma-Aldrich) diluted 1:25,000. Peroxidase-conjugated, species-appropriate antibody at a 1:5000 dilution was added and then detected by autoradiography using enhanced chemoluminescence (Merck Millipore). ACTB served as the loading control.

### Scratch wound healing migration assay

MSCs (passages 3–6) were seeded at a density of 1 × 10^4^ cells/cm^2^ in a 6-well plate and allowed to grow to confluence before being scratched with a P1000 pipette tip. Cell debris was removed by washing once with 1 ml of culture media. New culture media supplemented with 20 μM DH or 10 μM LPA was then added, and cells were maintained for up to 7 days concurrently with non-treated cells. The culture medium was changed every other day. Images of the closing wound were acquired on days 3, 5, and 7 by inverted microscopy. Three independent experiments were performed.

### Transwell migration assay

The MSCs were treated with 20 μM DH or 10 μM LPA for 24 h before being seeded into the insert chamber of an 8-μm pore size transwell (Corning) filled with DMEM supplemented with 2% (v/v) FBS, 100 U/ml penicillin, and 100 μg/ml streptomycin. The lower chamber contained 600 μl of completed DMEM medium (10% FBS). The culture was then maintained at 37 °C in a humidified atmosphere containing 5% CO_2_ for 6 h to allow cell migration. After 6-h incubation, the numbers of cells that migrated to the other side of the transwell inserts were determined by Hoechst-33342 staining. Data are presented as the mean ± standard error of the mean (SEM) of three independent experiments.

### Osteogenic differentiation

Cells at passages 3–6 were used to study osteogenic differentiation capacity. For osteogenic differentiation, MSCs at a density of 5 × 10^4^ cells were plated into a 35-mm tissue culture dish. NH OsteoDiff® Medium (Miltenyi Biotec, Bergisch Gladbach, Germany) was then added for osteogenic differentiation induction. After 2 weeks of culture, the cells were fixed with 4% formaldehyde for 15 min and stained with 40 mM Alizarin red S (Sigma Aldrich) for 20 min at room temperature (RT) to evaluate calcium deposition in the cells. Alizarin red S staining was quantitated by adding 10% acetic acid to the cells with subsequent incubation at RT for 30 min with shaking. The cells in 10% acetic acid were collected using a cell scraper, transferred into a 1.5-ml microcentrifuge tube, and incubated at 85 °C for 10 min. The cell mixture was centrifuged for 15 min at 20,000*g*. The supernatant was collected, and the pH value was adjusted to 4.1–4.5 with 10% ammonium hydroxide. Sample aliquots of 50 μl/well were prepared in triplicate in a 96-well plate to read the absorbance at 405 nm on a spectrophotometer (BioTek Instruments, Inc., Winooski, VT, USA). Alizarin red S deposited in the cells was calculated from the concentration of Alizarin red S relative to a standard curve.

### Adipogenic differentiation

For adipogenic differentiation, MSCs at passages 3–6 were used; 5 × 10^4^ cells of MSCs were cultured in NH AdipoDiff® Medium (Miltenyi Biotec) for 3 weeks. Cells were stained with 0.5% (w/v) Oil Red O (Sigma Aldrich) in isopropanol for 30 min at RT to determine the lipid droplet in the cells. For the quantification of Oil Red O staining, the dye was eluted with 100% isopropanol by incubating cells with isopropanol for 10 min at RT. Solution aliquots of 200 μl/well were transferred into a 96-well plate to read the absorbance at 510 nm by spectrophotometer (BioTek). Oil Red O concentration was calculated relative to a standard curve.

### Sample preparation for imaging by FTIR

Cells were trypsinized into single cells, and a drop of 4 × 10^5^ cells was deposited onto IR transparent 2-mm-thick barium fluoride windows, air dried, and washed several times with distilled water to eliminate culture medium contamination before storage in a desiccator until spectra were acquired.

### FTIR microspectroscopy analysis

FTIR spectra comprises three important regions, including the protein region (1700–1500 cm^−1^: amide I and amide II protein), the lipid region (3000–2800 cm^−1^: CH stretching, C=O ester lipid (1750–1700 cm^−1^, and the carbohydrate and nucleic acid region (1300–900 cm^−1^: P=O phosphates, C-O, C-C glygogen, and carbohydrate (1300–900 cm^−1^). In this study, spectrum data were acquired at an infrared microspectroscopy beamline (BL4.1 IR Spectroscopy and Imaging) at the Synchrotron Light Research Institute with a Vertex 70 FTIR Spectrometer (Bruker Optics Ltd., Ettlingen, Germany) coupled with an IR microscope (Hyperion 2000; Bruker Optics Ltd.). The detector of the infrared microscope was a liquid nitrogen-cooled mercury cadmium telluride (MCT-A) detector (of 100 μm in size). Measurements were performed using an aperture size of 10 × 10 μm with a spectral resolution of 6 cm^−1^, with 64 scans co-added over the measurement range from 4000 to 800 cm^−1^. Spectral acquisition and instrument control were performed using OPUS 7.5 software (Bruker Optics Ltd). Spectral changes in the functional groups were evaluated at the integral area of each peak, especially the region of amide I protein (1700–1600 cm^−1^), amide II protein (1600–1500 cm^−1^), CH stretching from lipid (3000–2800 cm^−1^), C=O ester lipid (1750–1700 cm^−1^), and PO43− or nucleic acid (1200–900 cm^−1^). Spectra from each sample group were analyzed using principal component analysis (PCA). Data were preprocessed by performing a baseline correction, and they were normalized using extended multiplicative signal correction using spectral regions from 3000 to 2800 cm^−1^ and 1800–900 cm^−1^ using Unscrambler 10.1 software (CAMO Software, Oslo, Norway).

## Results

### Effect of pharmacological molecules on YAP

**Fig. 1:**
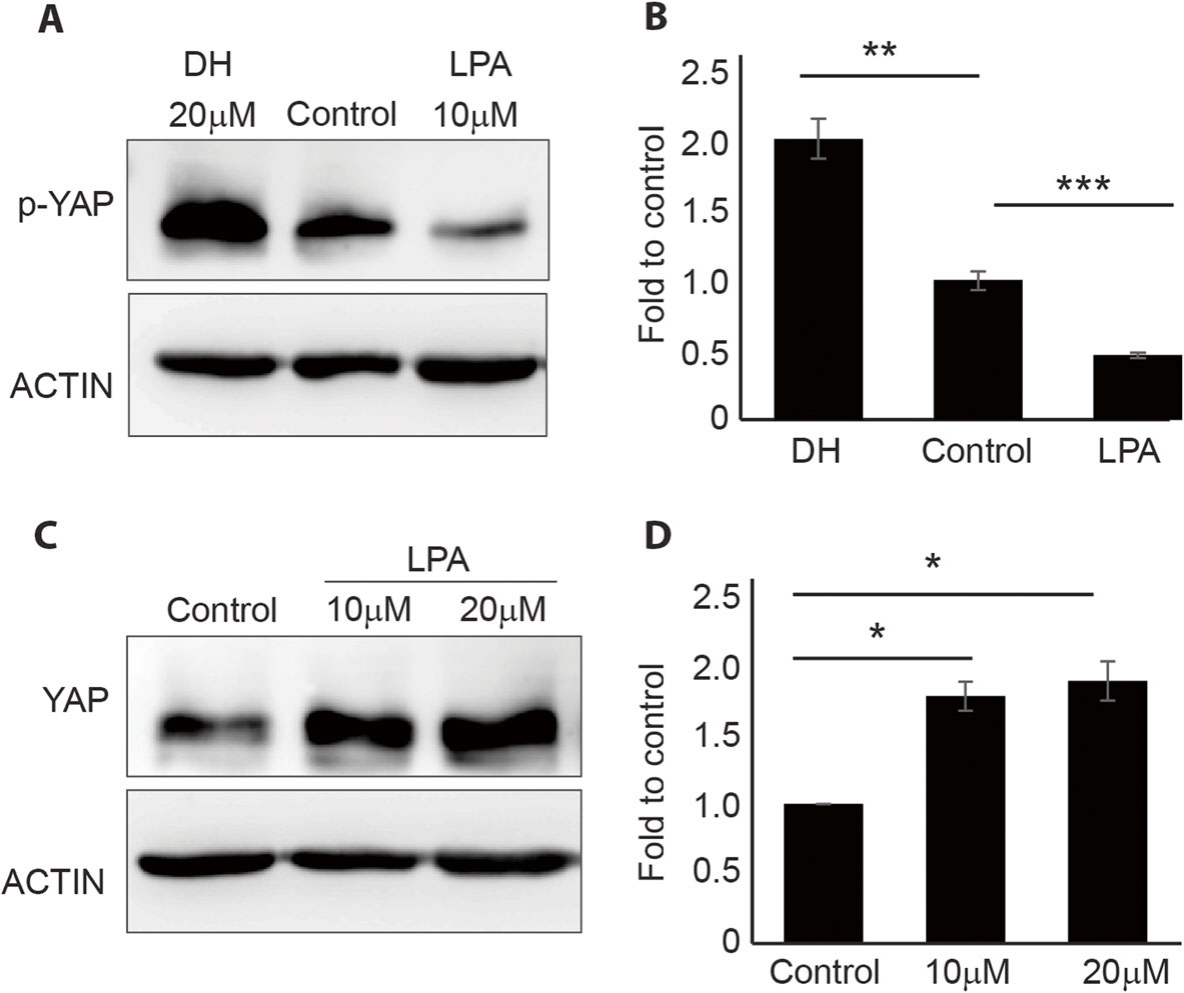
Effects of DH and LPA on the expression of YAP. Western blot analysis showed the phosphorylated form of YAP in MSCs after treatment with 20 μM DH or 10 μM LPA (**a**). Quantification of p-YAP protein levels confirmed a significant increase in p-YAP expression in DH and decreased p-YAP expression in LPA-treated cells (**b**). An increase in YAP upon treatment with LPA (**c**) and quantification of YAP protein levels confirmed a significant increase in YAP upon LPA treatment (**d**). Band intensity was normalized to that of actin (mean ± SEM; *n* = 3, *P < 0.05, **P < 0.01, ***P < 0.001, Student’s *t* test)

MSC samples that were characterized by immunophenotypic profiling (Additional file 1: Figure S1) were used in this study. To determine whether YAP was expressed in MSCs, we first determined the expression of YAP across cell types, including iPSCs, HEK293, and MSCs (Additional file 2: Figure S2). The results showed YAP to be highly expressed in MSCs and that it could be used as a model for studying the effect of small molecules on MSC differentiation. To confirm the efficacy of the pharmacological molecules, cells treated with 20 μM DH or 10 μM LPA were harvested for Western blot analysis to determine YAP activity. An increase in phosphorylated YAP (p-YAP; inactive form) was observed in DH-treated cells (Fig. 1a, b), and an increase in YAP active form was found in LPA-treated cells (Fig. 1c, d). These results indicated that the pharmacological molecules were working in the expected and appropriate manner.

### Effect of DH and LPA on human MSC proliferation and migration

**Fig. 2:**
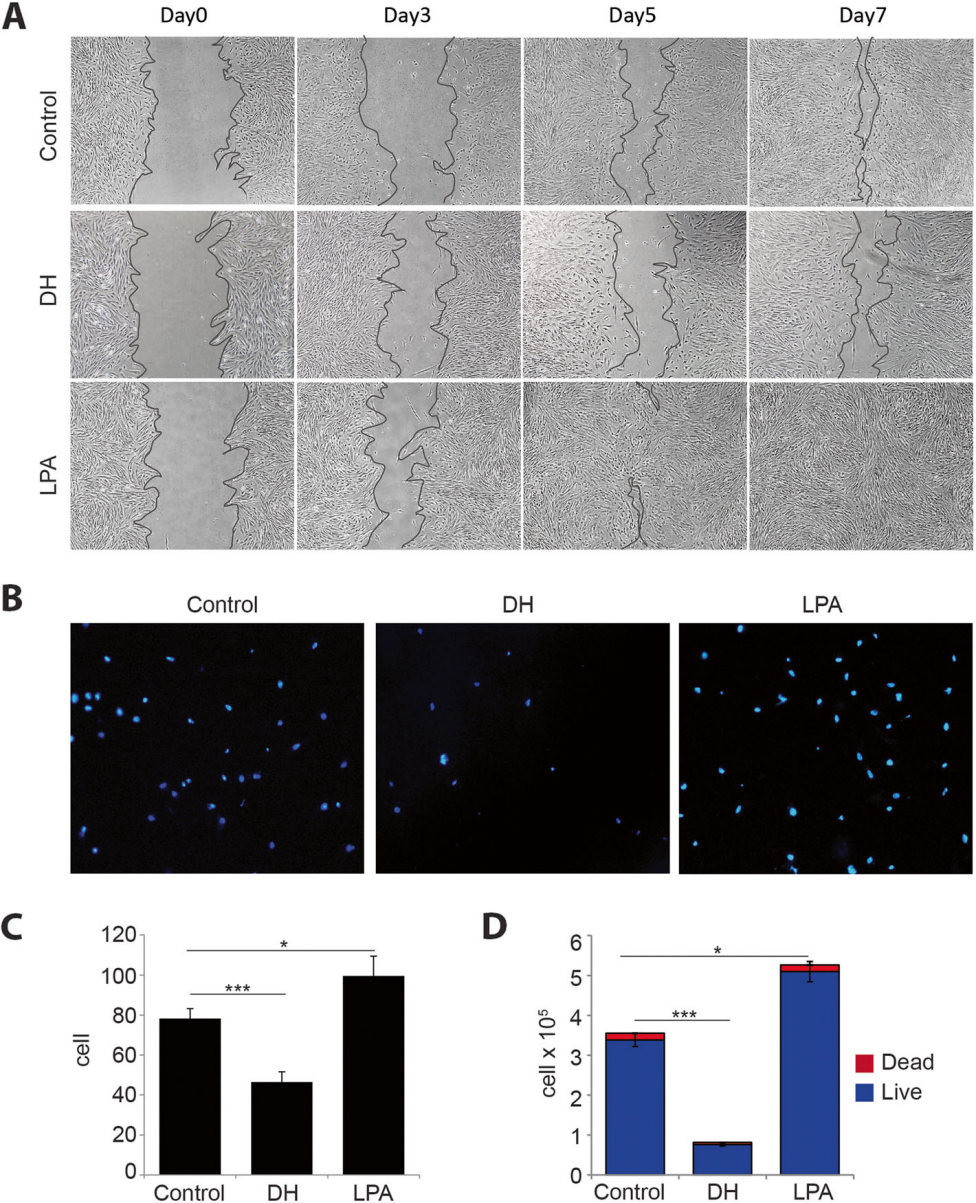
Effects of DH and LPA on MSC proliferation and migration. A scratch was made using a P1000 pipette tip and cultured in the presence of DH or LPA for 7 days. The distance between edges was monitored under a microscope to determine the proliferation rate upon treatment (**a**). Representative pictures of cells at the bottom side of the inserted chamber after 6-h incubation stained with Hoechst-33342 (**b**). The number of migrated cells was counted and reported as mean ± SEM; *n* = 3, *P < 0.05, ***P < 0.001, Student’s *t* test (**c**). MSC numbers were enumerated after treatment with DH and LPA with trypan blue exclusion (**d**). Statistical analysis for live cells is shown as mean ± SEM; *n* = 3, *P < 0.05, ***P < 0.001, Student’s *t* test. Numbers of dead cells were not significantly different among treatments

To determine whether pharmacological molecule treatment affects hMSC proliferation and migration, in vitro wound healing assay and transwell migration assay were performed. The result of the wound healing experiment showed that the wound area completely sealed on day 7 in the LPA-treated group, while cells treated with DH showed delayed effect compared to control (Fig. 2a). To further confirm that DH suppresses cell migration, we performed transwell migration assay. The result showed that DH-treated cells barely migrated to the bottom side, whereas LPA was found to enhance migration (Fig. 2b). Cell count analysis confirmed that a significantly less number of cells migrated to the bottom side of the upper chamber, as shown in Fig. 2c. Taken together, these results suggest that DH suppresses cell proliferation and migration, whereas the treatment of cells with LPA showed effects opposite to those treated by DH. To determine whether DH treatment induces cell death, cells at day 5 of treatment were collected for total cell count and to determine the number of living and dead cells using trypan blue exclusion assay. We found a significantly increased number of cells (living + dead cells) in the LPA-treated group compared to other conditions, whereas the DH-treated group had a significantly lower total number of cells. However, the number of dead cells did not differ significantly among treatments (Fig. 2d). These results indicate that DH inhibits MSC proliferation and migration, but it does not significantly induce cell death.

### DH inhibits osteogenic differentiation and promotes fat-forming process of human MSCs, but the inverse was observed in LPA

**Fig. 3:**
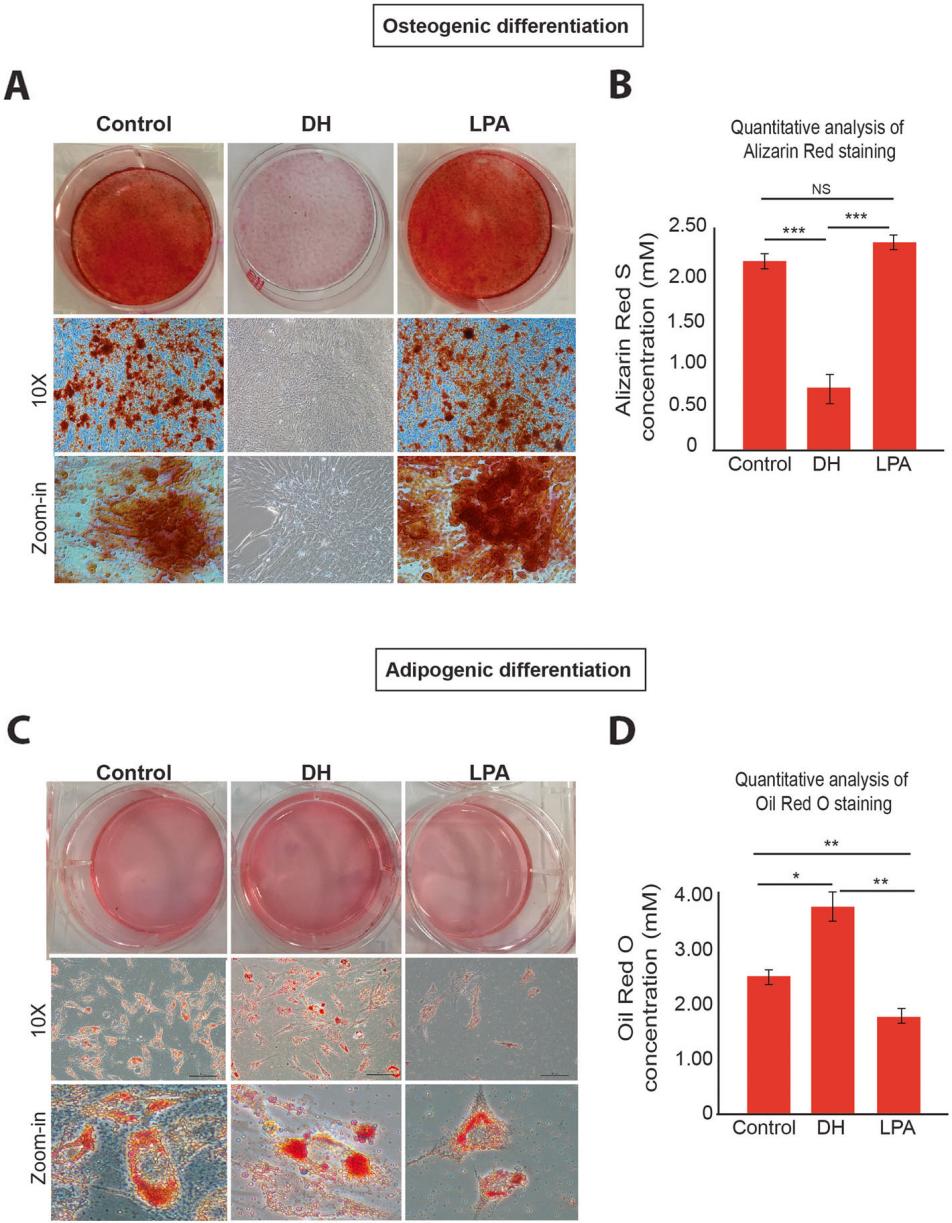
Effects of DH and LPA on osteogenic and adipogenic differentiation. Alizarin red staining shows calcium content (**a**) and quantitative determination of Alizarin red staining (**b**) in MSCs after treatment with LPA or DH. Oil Red O staining shows lipid droplets in cells after treatment with LPA or DH (**c**) and quantitative data (**d**). Quantitative data are presented as mean ± SEM; *n* = 3, *P < 0.05, **P < 0.01, ***P < 0.001, Student’s *t* test. NS, no significant difference

To determine whether the pharmacological molecules DH and LPA influence the differentiation capacity of MSCs, we performed osteoblast-like cell differentiation by culturing MSCs in cytokine-induced osteogenic differentiation medium in the presence of either LPA or DH (Fig. 3a). After 2 weeks of culture, we found that DH treatment inhibited the osteogenic differentiation of MSCs, while LPA promoted MSC differentiation to osteoblast-like cells, as demonstrated by Alizarin red staining (Fig. 3a). Quantitative measurement of calcium deposition from Alizarin red staining showed a significant decrease in calcium content in DH-treated MSCs (Fig. 3b). These results suggest that the expression level of YAP, which is a target protein of DH and LPA, could influence the differentiation capacity of MSCs.

The effects of pharmacological molecules were also investigated during the fat-forming process of MSCs. In contrast to osteogenic differentiation, DH-treated cells showed increased intracellular fat droplets compared to both control and LPA-treated cells (Fig. 3c). Quantitative measurement of fat droplets from Oil Red O staining showed a significant increase in fat content in DH-treated MSCs (Fig. 3d). These results suggest that the expression of YAP influences the differentiation of human MSCs.

### Gain- and loss-of-function experiments confirm the role of YAP in adipo-osteogenic differentiation

**Fig. 4:**
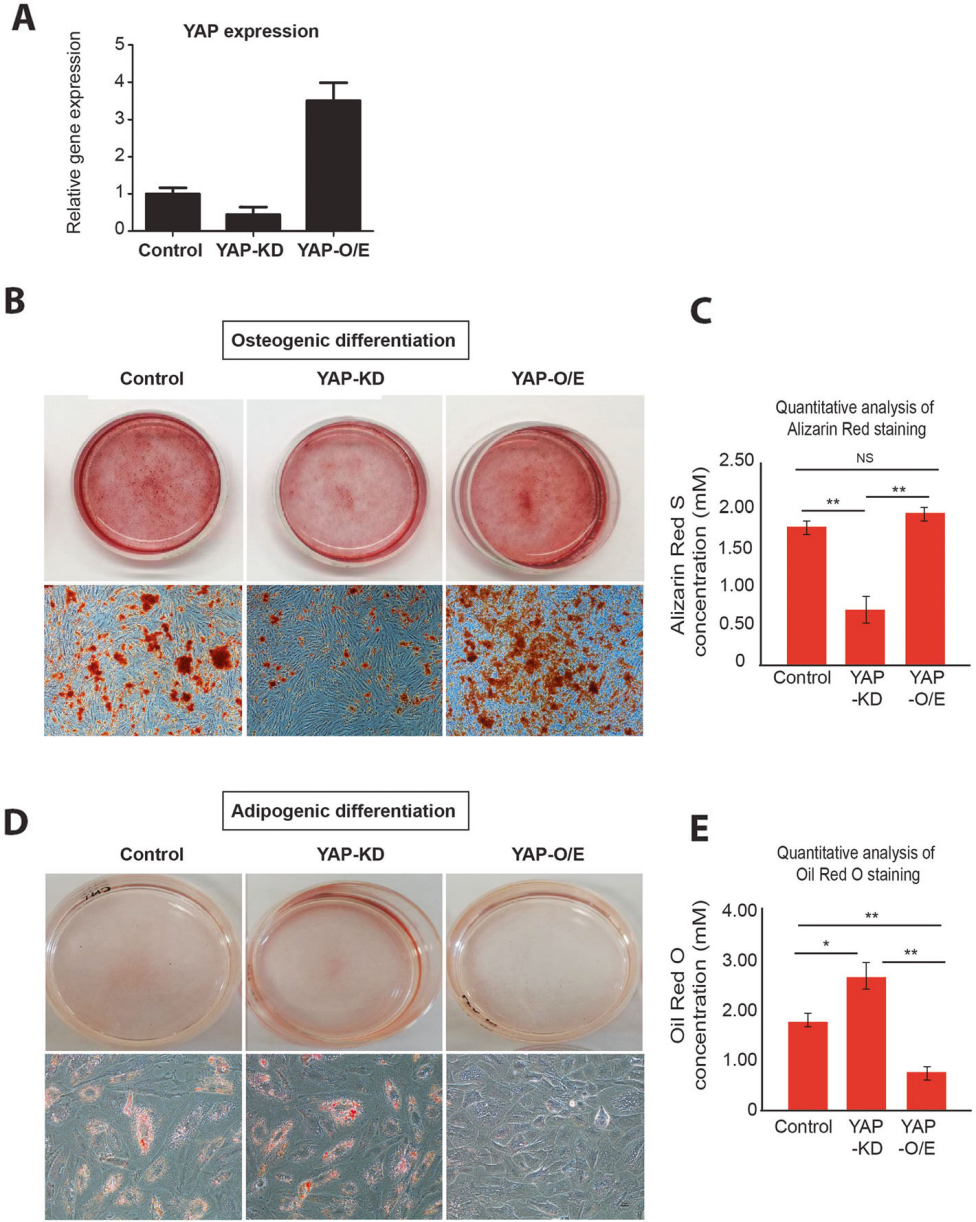
Effects of gene manipulations on *YAP* relative to osteogenic differentiation. *YAP* expression level in MSCs after being forced to be downregulated and upregulated as quantitated by q-PCR (**a**). Alizarin red staining shows calcium content and quantitative evaluation of Alizarin red staining in control, YAP-KD, and YAP-O/E MSCs (**b**, **c**). Oil Red O staining shows lipid contents and quantification value (**d**, **e**). Quantitative data are shown as mean ± SEM; *n* = 3, *P < 0.05, **P < 0.01, Student’s *t* test. NS, no significant difference

To further confirm that YAP plays a critical role in osteogenic differentiation, we performed gain- and loss-of-function experiments by generating YAP-knockdown (YAP-KD) and YAP-overexpressing (YAP-O/E) MSCs. The knockdown and overexpression efficiency rates were determined, as shown in Fig. 4a. Later, YAP-KD and YAP-O/E MSCs were subjected to osteogenic and adipogenic differentiation medium. Consistent with the results of small molecule treatment, YAP-KD cells exhibited less osteogenic differentiation phenotype when compared to those of YAP-O/E and control cells (Fig. 4b, c). In contrast, YAP-depleted cells demonstrated an increased bias towards adipogenic lineage commitment than YAP-overexpressing cells, as shown in Fig. 4d, e. These results confirm that YAP plays a crucial role in both adipogenic and osteogenic differentiations.

### FTIR signatures of YAP-depleted MSC-derived adipocytes and osteoblasts

**Fig. 5:**
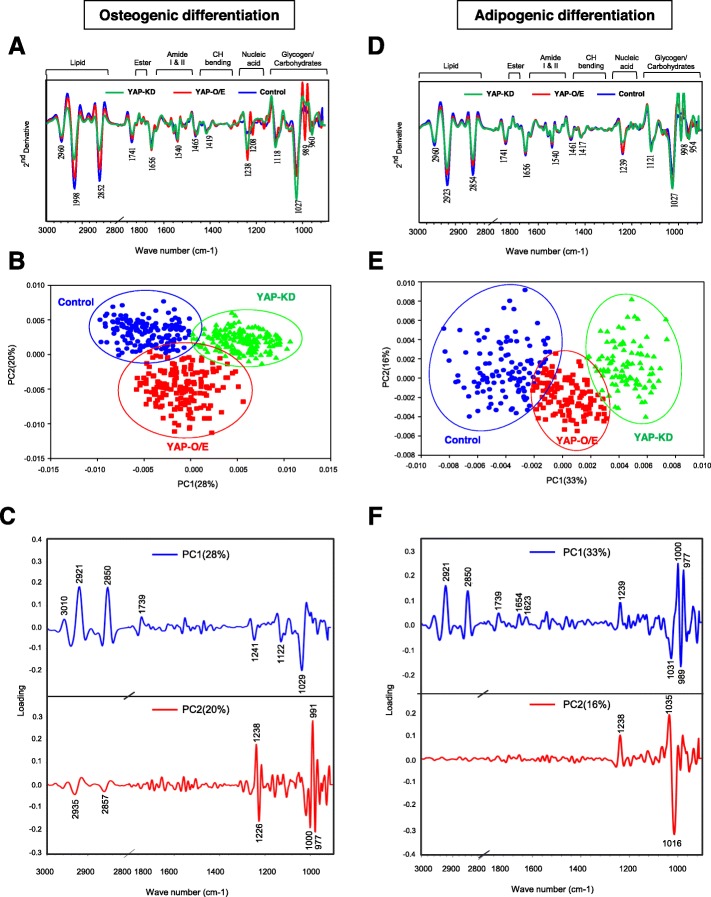
FTIR signatures of YAP-depleted MSC-derived adipocytes and osteoblasts. Second derivative spectral (**a**, **d**), 2D PCA score plot of all recorded FTIR spectra of YAP-O/E, YAP-KD, and control cells (**b**, **e**). Score loading of PC1 (upper) and PC2 (lower) to identify the variable corresponding to wavelength number (**c**, **f**). Blue dots represent non-treated control, green triangles represent YAP-KD, and red squares represent YAP-O/E cells. Eclipses depicted in the plot define the confidence level with which 95% of the data are allocated

To further confirm that the expression of YAP could influence the differentiation potential of hMSCs to bone and fat, differentiated cells were collected and subjected to FTIR analysis. The mean FTIR spectra, PCA analysis, and loading information of the control MSCs, YAP-O/E, and YAP-KD cells differentiated to adipocytes and osteoblasts recorded from more than 200 single cells in the mid-IR region of 4000–800 cm^−1^ are shown in Fig. 5. For differentiation to osteoblasts, PCA was performed on the second derivative spectra from control MSC, YAP-O/E, and YAP-KD (Fig. 5a). Three groups of spectra were clearly separated into 2D PCA score plots: PC1 and PC2 (Fig. 5b). PC1 and PC2 explained 28% and 20% of the total variance, respectively. The spectrum groups of YAP-KD were associated with positive scores on the score plot (PC1) and presented as a negative loading of PC1. The high negative loading for PC1 loading at 1029, 1122, and 1241 cm^−1^ assigned to PO_4_^3−^ phosphate bands, and nucleic acid from phosphodiester bonds (centered at 1241 cm^−1^) was responsible for distinguishing the YAP-KD cells from control and YAP-O/E cells (Fig. 5c). The YAP-O/E group was clearly separated along the negative score plot of PC2. The negative value of PC2 loading at 2931 and 2857 cm^−1^ corresponding to the C–H stretching bands, 1226 cm^−1^ associated with phosphodiester bonds from the nucleic acid, and PO_4_^3−^ phosphate bands centered at 977 cm^−1^ and 1000 cm^−1^ (Fig. 5c).

Unique FTIR spectra were also found in adipogenic differentiation. The second derivative spectra from control MSC, YAP-O/E, and YAP-KD cells that differentiated to adipocytes are shown in Fig. 5d. The PCA score plot demonstrated the separation of these samples with a total variance of 33% for PC1 and 16% for PC2 (Fig. 5e). The highest positive loading plot from PC1 was observed in the CH stretching (CH2, CH3 stretching) centered at 2921 and 2850 cm^−1^ from lipid, and C=O ester lipid centered at 1741 cm^−1^, and oppositely correlated with negative score plot in the MSC group from the second derivative spectrum (Fig. 5f). The separation along PC2 can be explained by the positive loading for PC2 in the spectral region at 1238 and 1035 cm^−1^ from the nucleic acid and carbohydrate regions, respectively. The respective spectra of the YAP-O/E were associated with negative score plots in PC2 and presented as a positive loading of PC2 (Fig. 5f). Taken together, these results show that FTIR signatures could clearly demonstrate the consequence of YAP upon adipo-osteogenic differentiation. Similarly, unique FTIR spectra were also observed in LPA- or DH-treated cells differentiated to osteoblasts and adipocytes (Additional file 3: Figure S3).

## Discussion

YAP has been implicated in several types of cells for controlling cell proliferation and differentiation [29, 30]. However, whether YAP plays a role in controlling adipo-osteogenic balance in humans has never been fully elucidated. In the present work, we showed that the expression level of YAP during human MSC differentiation is crucial for adipo-osteogenic differentiation. Increasing YAP, either by pharmaceutical molecule or by genetic manipulation, enhances osteogenic differentiation but suppresses differentiation to adipocytes even though the cells were cultured in enriched cytokine medium that promotes and supports adipogenic differentiation. In contrast, low YAP promotes adipogenic differentiation but inhibits osteogenic differentiation. These results clearly suggest that YAP plays a crucial role in human adipo-osteogenic differentiation.

YAP is highly expressed in highly proliferative cells, such as iPSCs and cancers, since it plays a role in inducing cell proliferation and anti-apoptosis [31, 32]. Even though the Hippo-YAP/TAZ signaling pathway has been studied in pluripotent stem cells for quite some time, the role of YAP in pluripotent stem cells remains inconclusive and controversial. A study in mouse embryonic stem cells (mESCs) found that YAP is highly expressed in self-renewing mESCs but is inactivated during differentiation [33]. Overexpression of YAP inhibits mESC differentiation and maintains stem-like properties and self-renewal even under differentiation conditions. In contrast, Chung and colleagues reported that YAP is dispensable for self-renewal, but it is required for differentiation [29]. Interestingly, a study in human pluripotent stem cells found YAP to be irrelevant relative to maintaining pluripotency but required for self-renewal. Overexpression of YAP could transform pluripotent stem cells into naive stem cells [30].

Aside from controlling the biological properties of pluripotent stem cells, YAP in MSCs seems to have no role in cell proliferation and anti-apoptosis processes. YAP was instead found to be crucial for the mechanosensing process by responding to the interaction of cell-matrix adhesion and by conveying the differentiation phenotype of MSCs [34–36]. In soft ECM, YAP retained in the cytoplasm undergoes a degradation process. This phenomenon enhances the likelihood of differentiation towards adipogenesis. Conversely, stiff ECM induces YAP to translocate into the nucleus to induce osteogenesis. These results clearly suggest that genes that are involved in adipo-osteogenesis are downstream targets of YAP. In this experiment, the expression of YAP was manipulated by pharmaceutical molecules and genetic approaches to determine the differentiation capacity of MSCs. Our results confirm that YAP is an essential molecule for adipo-osteogenic lineage decision of MSCs.

The mechanism by which YAP regulates adipo-osteogenesis was demonstrated by Pan and colleagues [17]. They found that YAP interacts with beta-catenin to promote osteogenic differentiation and maintain bone homeostasis in a mouse model [17]. Recently, transcriptional coactivator with PDZ motif (TAZ), a YAP homolog protein, was identified in mouse MSCs (C3H10T1/2 cell line) and in human adipose tissue-derived stem cells as an important effector protein that binds to Smad4 to regulate the balance of lineage commitment in osteogenic and adipogenic differentiation. However, they found that YAP did not interact with Smad4 in either osteogenic or adipogenic differentiation of MSCs [19]. Thus, multiple mechanisms may account for YAP regulation of adipo-osteogenesis of human MSCs, which requires further investigation.

FTIR microspectroscopy has been applied in the biomedical field to study global structural and compositional changes in the nucleic acids, proteins, and lipids of many biological samples, including stem cells and differentiated cells [24, 37–42]. SR FTIR was shown to provide information that can be used as a chemical fingerprint of biological cells. It is very useful for detecting physiological changes in cells that can be related to their physiological properties. Previous studies in stem cells have shown that the structures of nucleic acid, protein, and lipid have changed along the differentiation time course [24, 38, 41, 42] and that these changes result in a shift in the corresponding peaks in the FTIR spectra. Hence, the dynamics of the peaks during differentiation may have the potential for use as biomarkers to identify the stage of the cell. Moreover, FTIR microspectroscopy has a high signal-to-noise ratio of less than 10 μm per spot on the sample and enhanced diffraction-limited lateral spatial resolution. These advantages suggest the possibility that FTIR microspectroscopy can be used to identify even a small difference in a cell at the single-cell level. So far, this technique can be applied to study stem cell differentiation. The spectral signature of cells can be applied to create a spectral database of stem cell differentiation that will allow us to identify correlations between differentiation potential and spectral signature.

## Conclusions

In this study, we demonstrated that the expression level of YAP is essential for adipo-osteogenic differentiation of human MSCs. Increasing YAP activity, either by pharmaceutical molecule or by genetic manipulation, enhances osteogenic differentiation but suppresses differentiation to adipocytes. In contrast, low YAP activity promotes adipogenic differentiation but inhibits osteogenic differentiation. These results clearly suggest that YAP plays a crucial role in human adipo-osteogenic differentiation. We also showed that FTIR can be used as an effective method for studying cell characteristics of MSC-derived osteoblasts and adipocytes.

## Supplementary information

**Additional file 1:** **Figure S1.** UC-hMSCs were subjected to immunophenotypic profiling using flow-cytometry. Cells from all three cell lines (A-C) did not express CD34 or CD45, but they were positive for CD73, CD90, and CD105 expression. (PPTX 102 kb)

**Additional file 2:** **Figure S2.** Western blot analysis of total YAP and actin in induced pluripotent stem cells (iPSCs), HEK293, and MSCs.

**Additional file 3:** **Figure S3.** FTIR spectral signatures of MSCs treated with LPA or DH during differentiation towards osteoblasts or adipocytes. Second derivative spectral (A, E), two-dimensional PCA score plot of all recorded FTIR spectra of DH, LPA, and control cells (B, F). Score loading of PC1 (C, G) and PC2 (D, H) to identify the variable corresponding to wavelength number. Blue dots represent non-treated control, green triangles represent DH, and red squares represent LPA treated cells. Eclipses depicted in the plot define the confidence level with which 95% of the data are allocated. (PPTX 450 kb)

## Data Availability

All datasets in this article are included within the article and additional files.

## Abbreviations

MSCs: Mesenchymal stem cells
YAP: Yes-associated protein
UC: Umbilical cord blood
FTIR: Fourier transform infrared microspectroscopy
Runx: RUNX family transcription factor 2
PPAR: Peroxisome proliferator-activated receptor γ
PEPCK: Phosphoenolpyruvate carboxykinase
ALBP: Adipocyte lipid-binding protein
DH: Dobutamine hydrochloride
LPA: Lysophosphatidic acid
KD: Knockdown
O/E: Overexpression
