# Supplementary figures and images for: YAP as a key regulator of adipo-osteogenic differentiation in human MSCs
Source: Stem Cell Res Ther. 2019 Dec 18;10:402. doi: 10.1186/s13287-019-1494-4 (PMC6921580; doi:10.1186/s13287-019-1494-4)

## Slide 1
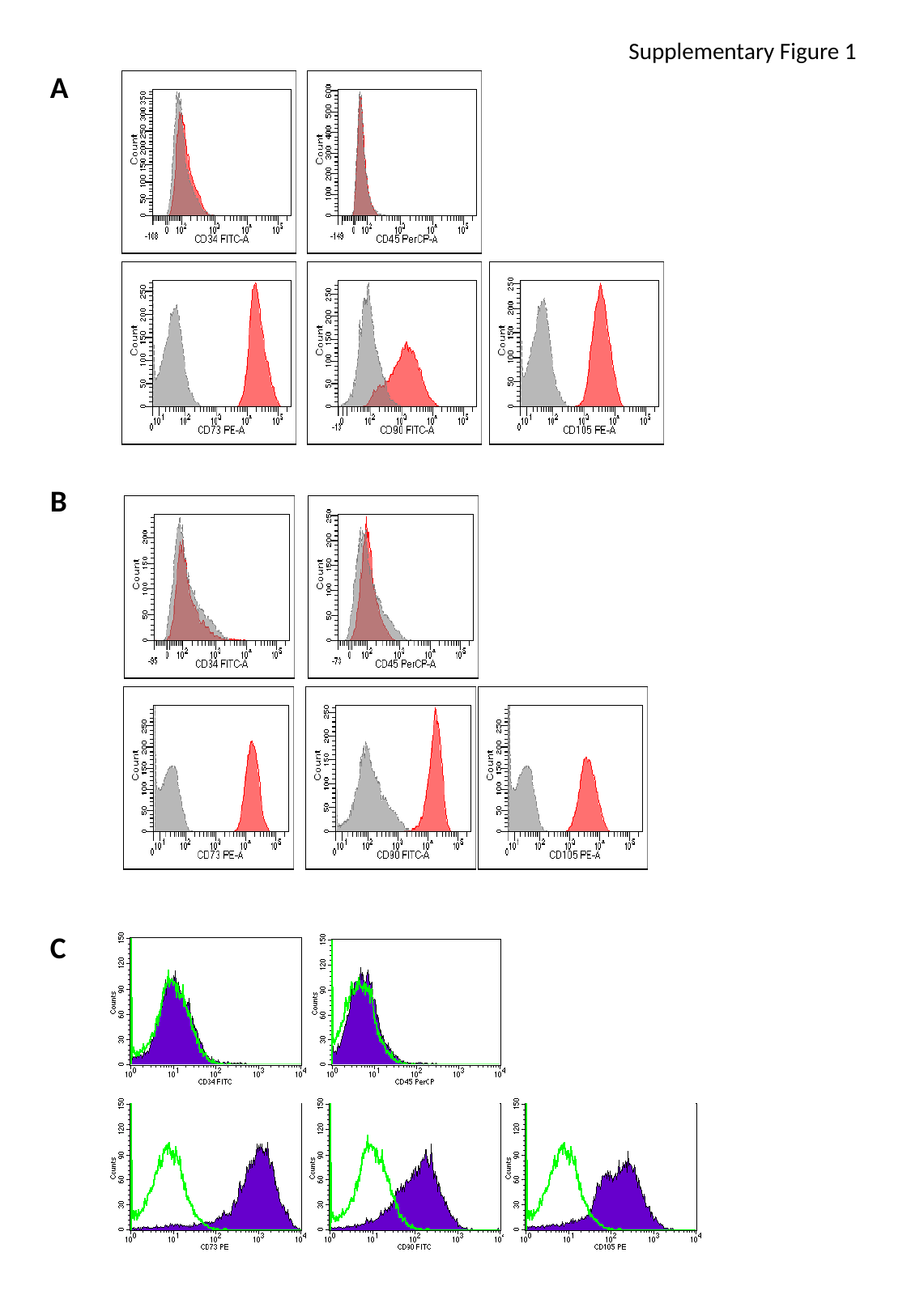

Supplementary Figure 1
A
B
C

Supplement: Supplementary file 1 — Additional file 1: Figure S1. UC-hMSCs were subjected to immunophenotypic profiling using flow-cytometry. Cells from all three cell lines (A-C) did not express CD34 or CD45, but they were positive for CD73, CD90, and CD105 expression. (PPTX 102 kb) [file 13287_2019_1494_MOESM1_ESM.pptx]

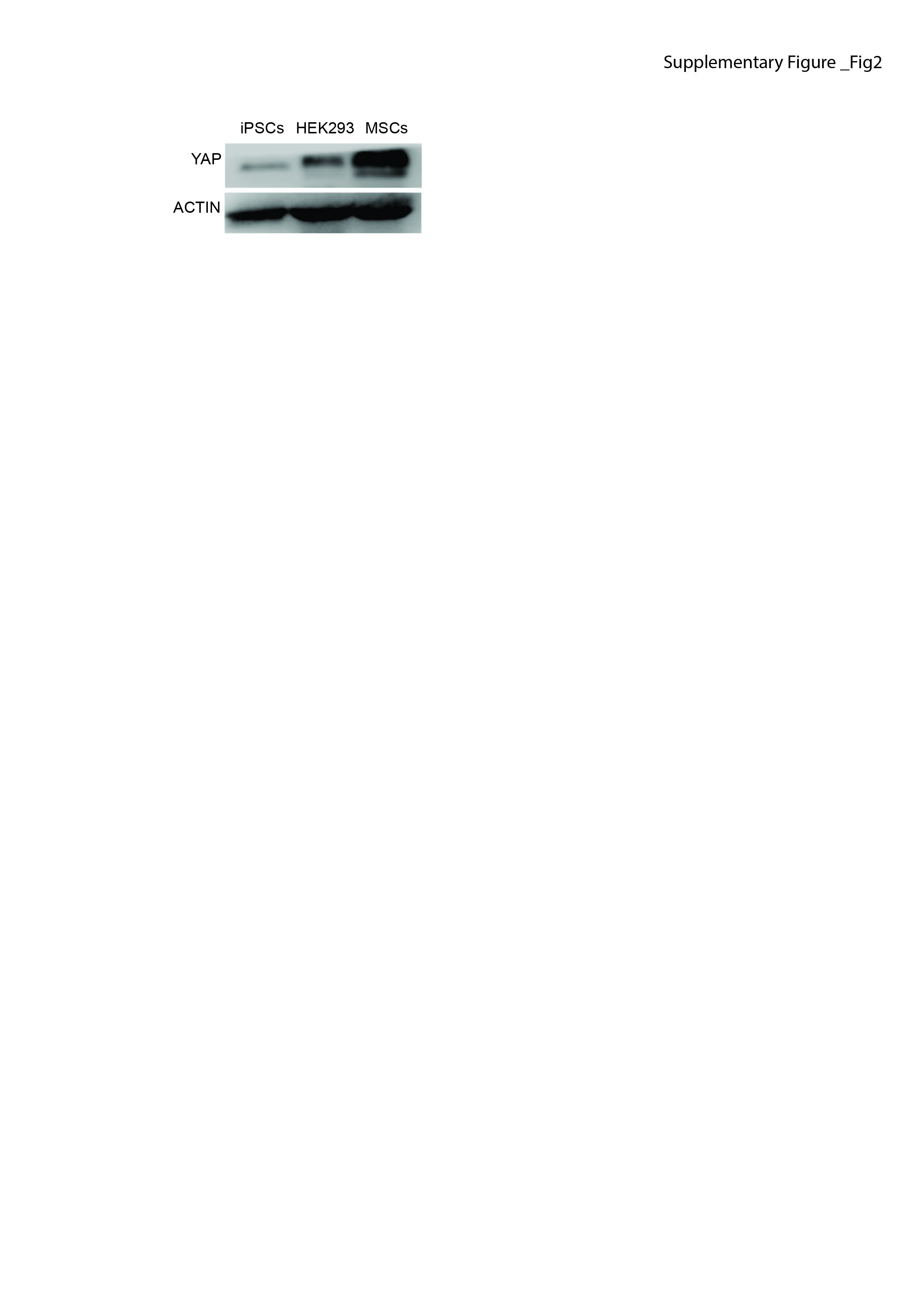

Supplement: Supplementary file 2 — Additional file 2: Figure S2. Western blot analysis of total YAP and actin in induced pluripotent stem cells (iPSCs), HEK293, and MSCs. [file 13287_2019_1494_MOESM2_ESM.jpg]
