# Supplementary material for: YAP as a key regulator of adipo-osteogenic differentiation in human MSCs
Source: Stem Cell Res Ther. 2019 Dec 18;10:402. doi: 10.1186/s13287-019-1494-4 (PMC6921580; doi:10.1186/s13287-019-1494-4)
Supplement: Supplementary file 3 — Additional file 3: Figure S3. FTIR spectral signatures of MSCs treated with LPA or DH during differentiation towards osteoblasts or adipocytes. Second derivative spectral (A, E), two-dimensional PCA score plot of all recorded FTIR spectra of DH, LPA, and control cells (B, F). Score loading of PC1 (C, G) and PC2 (D, H) to identify the variable corresponding to wavelength number. Blue dots represent non-treated control, green triangles represent DH, and red squares represent LPA treated cells. Eclipses depicted in the plot define the confidence level with which 95% of the data are allocated. (PPTX 450 kb) [file 13287_2019_1494_MOESM3_ESM.pptx]

## Slide 1
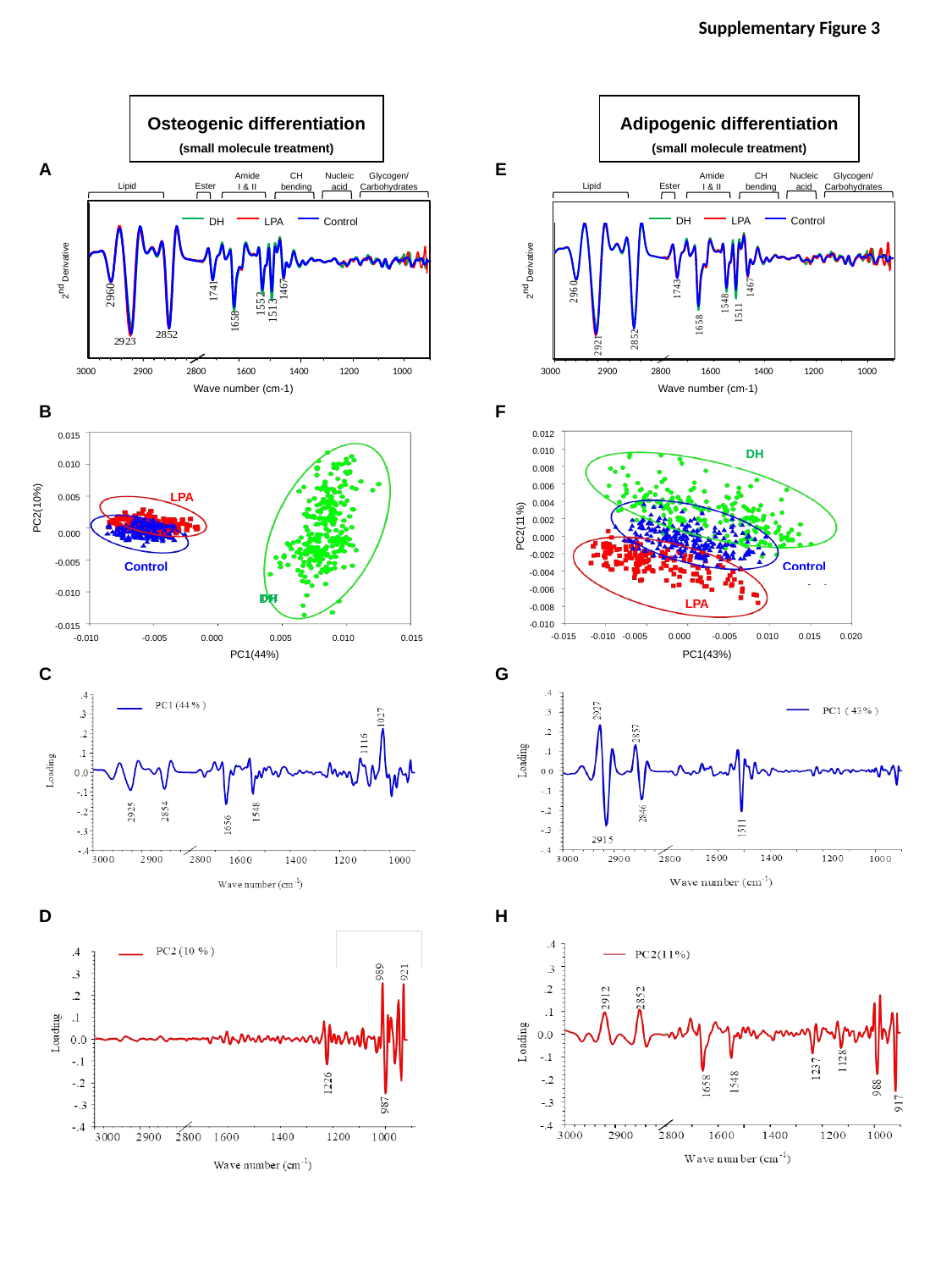

Supplementary Figure 3
Osteogenic differentiation
(small molecule treatment)
Adipogenic differentiation
(small molecule treatment)
A
E
Amide
I & II
CH bending
Nucleic
acid
Glycogen/
Carbohydrates
Lipid
Ester
Amide
I & II
CH bending
Nucleic
acid
Glycogen/
Carbohydrates
Lipid
Ester
DH
LPA
Control
DH
LPA
Control
2 Derivative
nd
2 Derivative
nd
3000
2900
2800
1600
1400
1200
1000
Wave number (cm-1)
3000
2900
2800
1600
1400
1200
1000
Wave number (cm-1)
B
F
0.012
0.015
0.010
0.005
-0.005
-0.010
-0.015
0.000
0.010
DH
0.008
0.006
LPA
0.004
PC2(10%)
0.002
PC2(11%)
0.000
-0.002
Control
Control
-0.004
-0.006
DH
LPA
-0.008
-0.010
-0.015
-0.010
-0.005
0.000
-0.005
0.010
0.015
0.020
-0.010
-0.005
0.000
0.005
0.010
0.015
PC1(44%)
PC1(43%)
C
G
D
H
